# Exploring the Plasticity of Diet on Gut Microbiota and Its Correlation with Gut Health

**DOI:** 10.3390/nu15153460

**Published:** 2023-08-04

**Authors:** Siqi Yao, Yiming Zhao, Hao Chen, Ruizheng Sun, Liyu Chen, Jing Huang, Zheng Yu, Shuijiao Chen

**Affiliations:** 1 Department of Gastroenterology, Xiangya Hospital of Central South University, Changsha 410008, China; ysqzj1999@163.com; 2 Department of Microbiology, School of Basic Medical Science, Central South University, Changsha 410078, China; yimingzhao970904@163.com (Y.Z.); sunruizheng98@csu.edu.cn (R.S.); chenliyu@csu.edu.cn (L.C.); 3 Department of Parasitology, School of Basic Medical Science, Central South University, Changsha 410078, China; chenho998@163.com (H.C.); jing_huang@csu.edu.cn (J.H.); 4 National Clinical Research Center for Geriatric Disorders, Xiangya Hospital of Central South University, Changsha 410008, China

**Keywords:** diet, gut microbiota, gut health, high protein, high fiber

## Abstract

Dietary habits have been proven to help alter the composition of gut microbiota, and exploring the impact of nutritional patterns on gut microbiota changes can help protect gut health. However, few studies have focused on the dietary impact on the gut microbiota over an experimental timeframe. In this study, 16S rRNA gene sequencing was employed to investigate the gut microbiota of mice under different dietary patterns, including AIN-93G diet (Control), high protein diet (HPD), high fiber diet (HFD), and switch diet (Switch). The alpha diversity of the HPD group significantly decreased, but HFD can restore this decline. During HPD, some genera were significantly upregulated (e.g., *Feacalibaculum*) and downregulated (e.g., *Parabacteroides*). However, after receiving HFD, other genera were upregulated (e.g., *Akkermansia*) and downregulated (e.g., *Lactobacillus*). In addition, the interaction between pathogenic bacteria was more pronounced during HPD, while the main effect was probiotics during HFD. In conclusion, the plasticity exhibited by the gut microbiota was subject to dietary influences, wherein disparate dietary regimens hold pivotal significance in upholding the well-being of the host. Therefore, our findings provide new ideas and references for the relationship between diets and gut microbiota.

## 1. Introduction

Dietary habits are an essential factor affecting the gut microbiota. The function of dietary changes in microbiota was first recognized more than a century ago [1]. Different dietary habits significantly influence the composition, diversity, and abundance of gut microbiota. The different dietary components will shape the gut bacterial community in a time-dependent manner [2]. The gut microbiota utilizes nutrients to grow and colonize in the intestine, and then the host maintains intestinal homeostasis through microbial metabolites, such as energy and immune modulators [3]. The gut microbiota can serve as an intermediate factor in intestinal inflammation. One of the mechanisms of dietary action on inflammation is that diet affects the composition and metabolic activity of the gut microbiota, leading to the ability of anti-inflammatory or pro-inflammatory functions [4].

Research had focused on the relationship between diet, gut microbiota, and host health. Diet alters the host’s metabolic pattern by regulating the balance of gut microbiota, ultimately affecting the immune system [5,6]. Currently, clinical nutrition is dedicated to treating autoimmune diseases through diet, aiming to target recombinant gut microbiota and restore intestinal barrier function [7]. Dietary habits can influence local bacterial changes by means of the nutritional components present in various diets. This can promote bacterial adhesion and infiltration, ultimately resulting in intestinal inflammation. [8]. Previous studies have demonstrated that a high fat diet and high salt diet led to a reduction in the intestinal slime layer [9]. Subsequently, the intestinal permeability and bacterial colonization rate on the intestinal mucosa increased, further promoting the inflammation [9]. Recent studies have pointed out that when mice were fed a high protein diet after dextran sulfonate (DSS) modeling, the slime layer became thinner and the degree of IBD was significantly increased. This is caused by a high protein diet altering the microbial composition of colon mucus [10]. In addition, a related study found that after intervention with high protein diet, the gut microbiota of mice showed an increase in *Helicobacter* and *Escherichia coli*, which was consistent with the performance of Crohn’s disease patients in the public database [11]. Therefore, we propose the promoting effect of a high protein diet on inflammatory bowel disease. However, recent studies have shown that a fiber diet plays a significant role in restoring the abundance, diversity, and composition of gut microbiota [12]. A study dedicated to exploring the relationship between dietary fiber and the risk of Crohn’s disease found that the intake of cellulose and zinc was negatively correlated with Crohn’s disease, especially the cellulose contained in fruits and vegetables [13]. This has been proven to be attributed to the specificity of soluble fibers in maintaining the intestinal barrier and preventing bacterial translocation, thereby reducing the occurrence of Crohn’s disease.

The research on dietary habits and gut microbiota mainly focuses on exploring the variations in gut microbiota after dietary intervention. However, there is no consensus on the specific alterations in gut microbiota during dietary interventions. In our study, we fed mice with AIN-93G diet (Control), high protein diet (HPD), high fiber diet (HFD), and switch diet (Switch) in mice. We aimed to explore the alterations in gut microbiota during the dietary intervention process through 16S gene sequencing, and to analyze the correlation between dietary patterns and intestinal health. Our research endeavors to establish a foundation for comprehending the impact of dietary habits on intestinal health and to offer novel references for the implementation of dietary therapy in clinical treatment.

## 2. Materials and Methods

### 2.1. Animal Experiments

According to the previous experimental design, female BALB/c mice (3 weeks old, 12–15 g) were purchased from Hunan Sleek Jingda (SLAC), Changsha, China [10]. All mice were raised in plastic cages covered with metal fences under specific pathogen-free conditions. The mice were housed in individually ventilated cages (IVCs) with free access to food and water. The environmental conditions of housing were maintained as follows: temperature (25 ± 5 °C), humidity (60–70%), and light (12/12 h light/dark cycle). Prior to commencing the experiment, mice were provided with a period of 5 days to adapt to the experimental environment.

**Figure 1:**
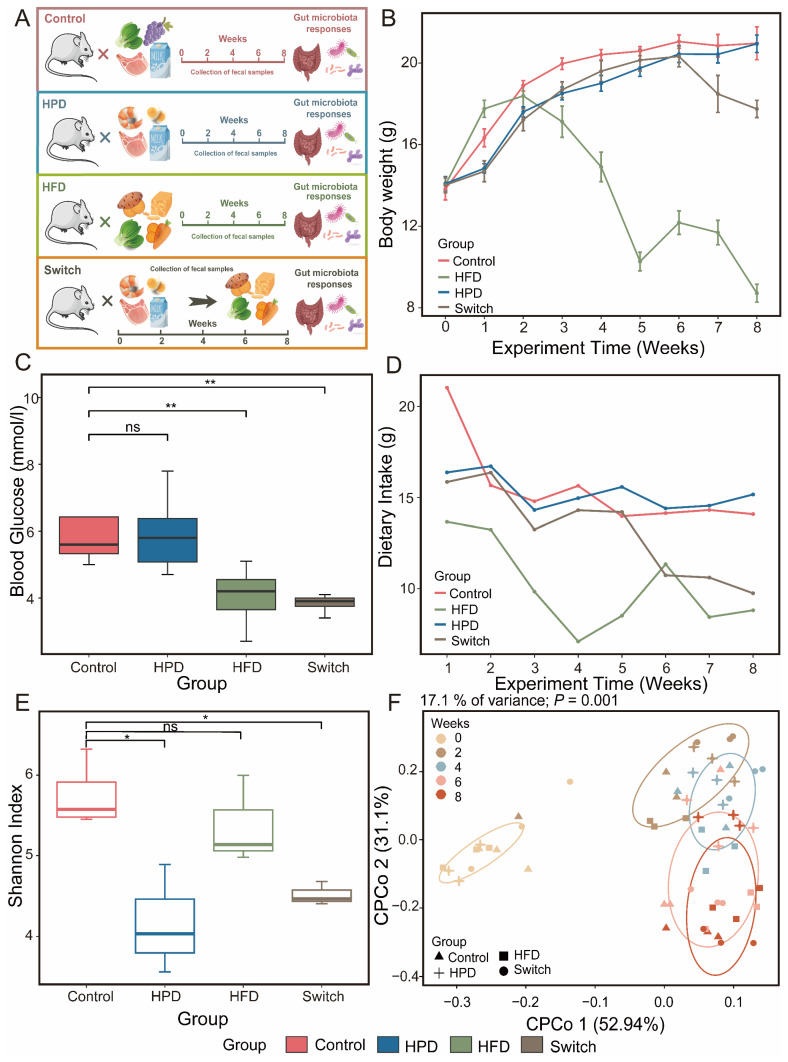
Experiment overview, mice characteristics, and microbial diversity in four groups. (**A**) The experimental design of this study. This study randomly divided mice into four groups: Control, HPD, HFD, and Switch. (**B**) Changes in body weight of mice in four groups throughout the experiment. (**C**) The box plot shows the differences in blood glucose levels among the four groups of mice at week 8 of this experiment. (**D**) Changes in weekly dietary intake of mice in four groups during whole experiment. (**E**) The alpha diversity of the four groups was compared using the Shannon index at week 8. (**F**) Analysis of CPCoA showcases the beta diversity of four groups. Each color represents a point in experimental time, and each shape represents a group (Wilcox. test, ns *p* > 0.05. * *p* < 0.05, ** *p* < 0.01).

After adaptive feeding, the mice were randomly divided into four groups with 8 mice in each group: Control group, HPD group, HFD group, and Switch group. Based on the experimental design, four groups were fed with different diets (Figure 1A). These diets were as follows: Control, healthy mice with AIN-93G diet; HPD, healthy mice with high protein diet; HFD, healthy mice with high fiber diet; Switch, healthy mice with high protein diet for four weeks followed by a high fiber diet for four weeks. All food was produced by Beijing HFK Bioscience Co., Ltd., Beijing, China. The production numbers were 20220249 and 20220274. The diet formulation for the Control group was complied with the American Institute of Nutrition’s food-grade ingredient formulation standard AIN-93G [14]. High protein and high fiber diets were modified according to AIN-93G criteria. The specific diet information of the four groups of mice was shown in Table 1. Furthermore, the percentages of nutrients and energy in the different dietary patterns were shown in Table 2. The study was approved by the Laboratory Animal Ethics Committee of Xiangya Hospital of the Central South University (No. 2022030735).

### 2.2. Sample Collection

Fecal samples of all mice were collected once every 2 days. After immobilizing the mice, lifted their tail and gently pressed the lower abdomen to collect fresh feces into sterile EP tubes, which were then stored at −80 °C for further analysis. The body weight of all mice and the dietary intake of four groups were measured once every 7 days. Blood glucose levels were measured in mice by collecting blood from the tail vein. After immobilizing the mice, the tail tip was gently trimmed to collect blood into a blood collection tube, which was subsequently used for glucose measurement. Blood glucose was measured before the mice were sacrificed at week 8. At week 8, after feeding, all mice were sacrificed by chloral hydrate asphyxiation and cervical dislocation. The collection of fecal samples and weight measurement were conducted one day before sacrifice. All mice were euthanized through cervical dislocation after the eighth week of dietary intervention.

### 2.3. DNA Extraction and High-Throughput Sequencing of 16S rRNA Gene

The DNA was extracted from fecal samples collected at different time points and the resulting qualified products were subjected to 16S rRNA gene sequencing. Bacterial DNA was extracted from stool samples using the DNeasy PowerSoil Kit (QIAGEN, Inc., Venlo, The Netherlands) according to the procedures described in the manufacturer’s instructions. A NanoDrop NC2000 spectrophotometer (Thermo Fisher Scientific, Waltham, MA, USA) and agarose gel electrophoresis were employed to measure the quantity and quality of DNA extracted. Then, total genomic DNA of all samples was stored at −20 °C for further analysis. The forward primers 338F (5′-ACTCCTACGGGAGGCAGCA-3′) and reverse primers 806R (5′- GGACTACHVGGGTWTCTAAT-3′) were used to amplify the DNA of the V3–V4 regions of the 16S rRNA gene. The polymerase chain reaction (PCR) was conducted under the following cycling conditions: initial denaturation at 98 °C for 2 min, followed by 25 cycles consisting of denaturation at 98 °C for 15 s, annealing at 55 °C for 30 s, and extension at 72 °C for 30 s, with a final extension of 5 min at 72 °C. The PCR products were purified with Agencourt AMPure Beads (Beckman Coulter, Indianapolis, IN, USA) and quantified using the PicoGreen dsDNA Assay Kit (Invitrogen, Carlsbad, CA, USA).

The qualified libraries were then sequenced on the Illumina NovaSeq platform with the NovaSeq-PE250 sequencing strategy at Shanghai Personal Biotechnology Co., Ltd. (Shanghai, China). After sequencing, the data were decomposed into appropriate samples based on barcodes and the appropriate sequences were imported into downstream software. The data were de-multiplexed into samples according to the barcodes, fitter sequences were imported to the Quantitative Insights into Microbial Ecology (QIIME2, 2022.2, https://qiime2.org/) [15]. Quality control and denoising of raw reads were performed based on the standard amplicon pipeline as described previously [16]. The feature table and taxonomy table were used for further data analysis. 

### 2.4. Data Analysis

All statistical analysis were conducted using R (version 4.2.1) [17]. The normality of the data was evaluated using the Shapiro–Wilk test, and the homogeneity of variance was assessed using the Bartlett test. If the results of both tests were *p* > 0.05, we selected the *t*-test for analyzing the differences. Otherwise, we chose the Wilcox test for analyzing the differences. Without any other instructions, all statistical results were visualized using the “ggplot2” package [18]. The Shannon index of alpha diversity and the Bray–Curtis distance of beta diversity were calculated using a “vegan” package [19]. Constrained principal coordinate analysis (CPCoA) based on Bray–Curtis distance was analyzed using the “Amplicon” package [20]. The Pearson correlation analysis was performed using the “corrplot” package [21]. Furthermore, biomarkers of sample groups were discovered by linear discriminant analysis (LDA) effect size (LEfSe) (http://huttenhower.sph.harvard.edu/galaxy/, accessed on 23 March 2023) [22]. The strategy for multi-class analysis was to set one-against-all, and the threshold on the logarithmic LDA score for discriminative features was set to 3.0, while the threshold of significance was set at 0.05. The “randomForest” package was used for random forest regression analysis [23]. The Spearman correlation analysis of genera with significant changes in relative abundance was calculated using the “Hmisc” package [24]. The interaction network between genera was visualized using the “igraph” package in the R [25] and Gephi software (version 0.10.0) (https://gephi.org/) [26]. The *p*-value threshold for error detection rate (FDR) correction by Benjamin and Hochberg was 0.05. The “pheatmap” package was used to visualize the relative abundance of genera, biomarkers, and correlation of genera [27].

## 3. Results

### 3.1. Effect of Dietary Habits on Physiological Characteristics

In this study, we aimed to investigate the impact of various dietary habits on gut microbiota (Figure 1A). We fed different diets to mice in the Control, HPD, HFD, and Switch groups. After receiving different diets, the weight of mice in the Control and HPD groups remained elevated. However, the weight of the mice in the HFD decreased after week 2 of the experiment. In the Switch group, the weight of mice decreased two weeks after receiving the high fiber diet (Figure 1B). It is noteworthy that after the experiment, we found that, compared to the Control group, the HFD and Switch groups presented a significant decrease in body weight (Figure S2A). This may be related to the dietary intake of mice. Monitoring the weekly nutritional consumption of four groups revealed that HFD had the lowest dietary intake (Figure 1D). The blood glucose levels of each group were measured in week 8 of this experiment. The results exhibited that it was similar to the Control group in the HPD group. However, both HFD and Switch showed a significant decrease in blood glucose levels (Figure 1C). 

### 3.2. Dynamic Changes in Microbial Diversity during Dietary Intervention

All sample diversity rarefaction curves tended to be parallel to the X-axis, indicating sufficient sequencing depth for all samples (Figure S1). Alpha diversity was represented by the Shannon index. We found that throughout the entire experimental process, the Shannon index of the Control, HPD, and Switch groups decreased first and then increased, but HFD displays the opposite trend (Figure S2B). In week 8 of the experiment, contrary to the Control group, we found a significant decrease in the diversity of HPD and Switch, but no significant difference in HFD. But compared to HPD, the alpha diversity of the Switch group has been restored (Figure 1E). The Bray–Curtis distance showed the beta diversity. The Bray–Curtis distances of the four groups increased consistently over the course of the experiment (Figure S2C). To further elucidate the effects of experimental time and dietary intervention on gut microbiota, we conducted PCOA analysis. Over the course of the experiment, the results demonstrated a gradual separation between samples on the second coordinate axis (Figure S3A). After dietary intervention, it was evident on the third coordinate axis that the distance between samples shifted under different dietary interventions (Figure S3B). Furthermore, based on the Bray–Curtis distance, CPCoA analysis showed that the distances between samples at each time point varied after receiving different diets and gradually increased (Figure 1F). The PCOA analysis demonstrated that intervention time and dietary differences are crucial factors in microbial changes.

### 3.3. Changes in Microbial Composition of Four Groups over Experimental Time

**Figure 2:**
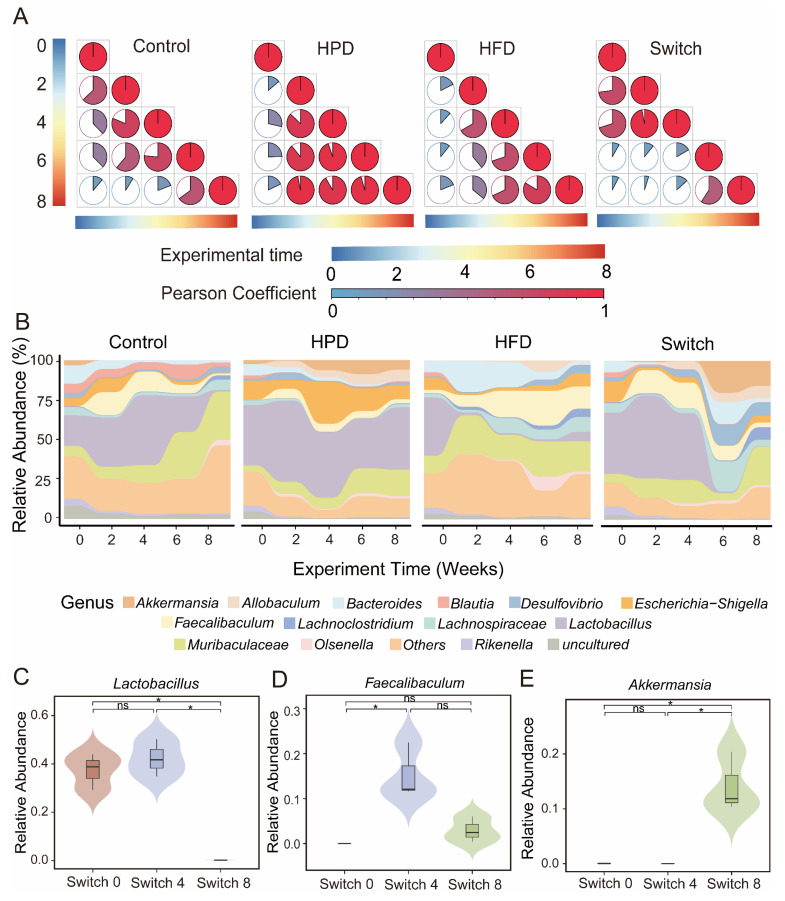
Dynamics of bacterial changes with experiment time of four groups. (**A**) Pearson correlation analysis showed a correlation between experimental time and microbial changes. Changes in the microbial structures in the Control, HPD, HFD, and Switch groups. The area and color of each pie chart represents the Pearson coefficients of two corresponding samples. (**B**) Bacterial community composition of Control, HPD, HFD, and Switch groups at genus level. Differences in abundance at the genus level over the course of experiment of the Switch group for *Lactobacillus* (**C**), *Feacalibaculum* (**D**), and *Akkermansia* (**E**). (Wilcox test, ns *p* > 0.05, * *p* < 0.05).

To further confirm the correlation between gut microbiota composition and experimental time after receiving different diets, we conducted a Pearson correlation analysis. The results showed that, in the Control group, the microbiota composition continued to decrease over the experimental time. In the HPD and HFD groups, the microbiota composition remained stable after two weeks of dietary intervention. In the Switch group, we observed a slow decline in the Pearson coefficient during the high protein diet period, followed by a rapid decline after switching to a high fiber diet (Figure 2A). This indicates rapid variations in the microbiota composition under different dietary interventions. In terms of microbial composition, we found commonalities and differences in the Control, HPD, HFD, and Switch groups. At the phylum level, Control, HPD, and HFD are chiefly composed of Firmicutes and Bacteroidetes (Figure S4A–C). The Switch group was dominated by Firmicutes, but Verrucomicorbiota also began to appear after receiving the high fiber diet (Figure S4D). To elucidate the differences in bacterial composition among each group, we selected the top 15 abundant genera to further investigate the changes in microbial composition of the 4 groups during the research duration (Figure 2B). At the genus level, the Control group displayed a decrease in *Lactobacillus* and an increase in *Muriaculaceae* throughout the experiment. The HPD group was mainly composed of *Lactobacillus* and *Escherichia-Shigella* during the complete investigation. The HFD group mainly included *Muribaculaceae* and *Feacalibaculum*. However, in the Switch group, we noticed that *Lactobacillus* were dominant in the process of high protein diet period, the composition of the gut microbiota rapidly changed after receiving a high fiber diet. Particularly, the relative abundance of *Lactobacillus* had decreased significantly. Based on the rapid transformation of the gut microbiota in the Switch group, we further analyzed the abundance of different bacteria at the genus level. *Lactobacillus* significantly decreased in week 8 of the experiment (Figure 2C). *Feacalibaculum* significantly increased in week 4 and decreased in week 8 (Figure 2D). *Akkermansia* significantly increased in week 8 (Figure 2E).

### 3.4. Critical Microbiota of Switch Group during Dietary Intervention

**Figure 3:**
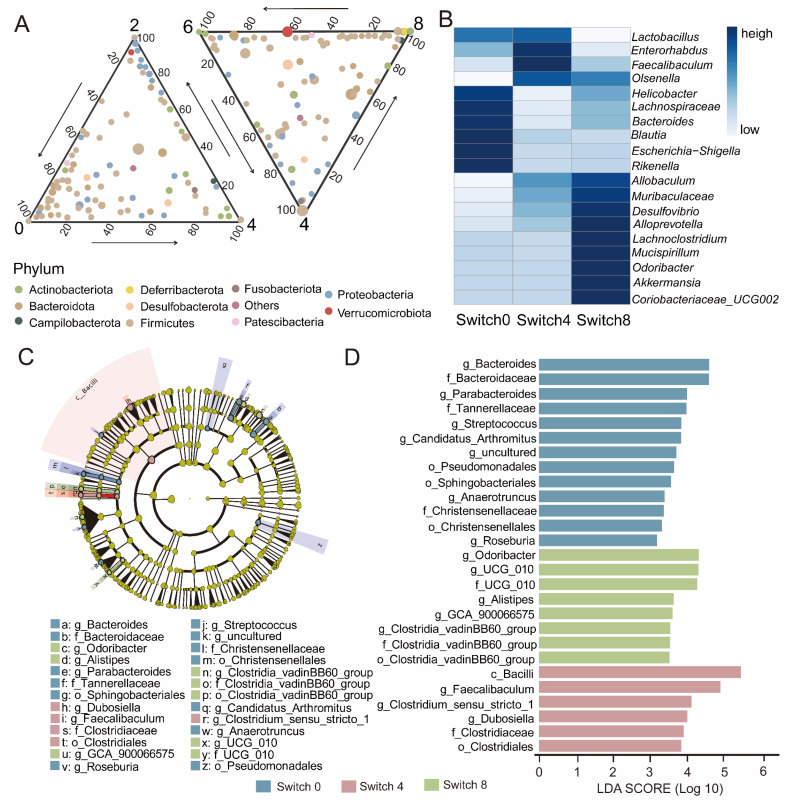
Changes in bacterial community dynamics of the Switch group over experiment time and LEfSe analysis of taxonomy with significant differences. (**A**) The ternary plot showed the changes in microbial composition of the Switch group at the genus level over experimental time. The size of each symbol represents its relative abundance and its color represents its phylum. (**B**) Heatmap showing the top 20 genera in terms of abundance in the Switch group. The color bar represents the relative abundance of the genus. (**C**) Evolutionary branching diagram. Regions with different colors represent different groups. Nodes in different colors represent significant changes in relative abundance in different groups. Yellow nodes indicate no significant changes in the corresponding group. (**D**) Histogram of LDA value. Taxa with significantly different abundances in different groups are shown, and the length of the bar graph represents the effect size of the significantly.

Due to rapid changes in microbial composition, we further divided the Switch group into three groups: Switch 0, before the experiment, Switch 4, after receiving a high protein diet, and Switch 8, after receiving a high fiber diet. To demonstrate the changes in the microbial community of the Switch group, the ternary plot showed that Proteobacteria clustered in the fourth week, while Verrucomimicrobiota increased in week 8 (Figure 3A). The heatmap further showed the variation in abundance of the top 20 genera in the Switch group (Figure 3B). We found high abundance in Switch 0 with *Blautia* and *Bacteroides*. The abundance of *Lactobacillus* and *Feacalibaculum* was higher in Switch 4 than in other groups. The abundance of *Akkermansia* and *Lachnoclostridium* increased in Switch 8. To provide further insight into the biomarkers of the Switch group at different dietary receiving stages, we used two methods to analyze the biomarkers of the Switch group at three stages. LEfSe analysis revealed that the identified biomarkers correspond to the previous inter-group differences at the genus level (Figure 3C). *Feacalibaculum*, *Alistipe*, *Bacteroides*, *GCA_ 900066575*, and *Dubiosiella* were key biomarkers. In Switch 0, *Bacteroides* and *Paraacteroides* were the most important bacteria. The abundance of *Feacalibaculum* and *Dubosella* significantly increased in Switch 4. *Odoribactor* and *GCA_ 900066575* were also increased in Switch 8 (Figure 3D). 

**Figure 4:**
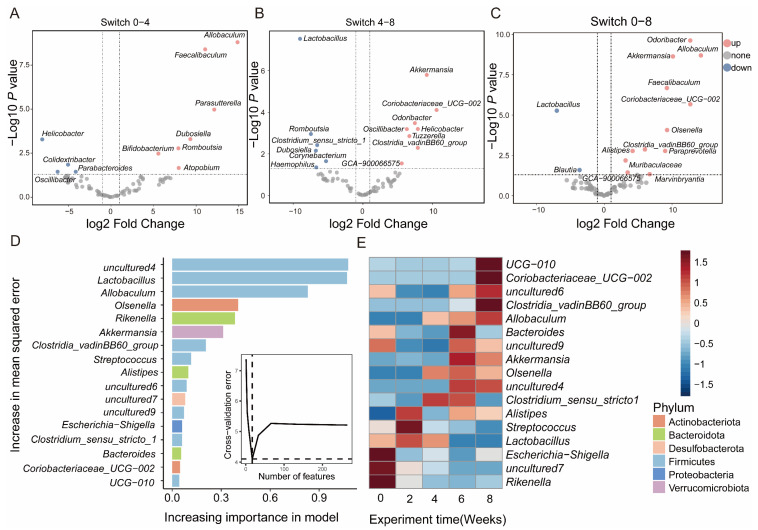
Differential bacteria of progression over time in the Switch group. The volcano plot shows significantly upregulated and downregulated genera in Switch 0 and Switch 4 (**A**); Switch 4 compared with Switch 8 (**B**); Switch 0 compared with the Switch 8 (**C**). The importance of differential bacteria in the Switch group through machine learning (**D**). The heatmap was used to show changes in key genera over the entire investigation period (**E**). The color bars represent correlations.

At the genus level, *Odoribactor*, *GCA_ 900066575*, and *Dubosella* showed significant differences in different groups (Figure S5A–C). In contrast with Switch 0, Switch 4 showed that *Feacalibaculum*, *Dubosella*, and *Romboutsia* were upregulated, while *Paraacteroides* and *Helicobacter* were downregulated (Figure 4A). Switch 8 exhibited an increase in *Odoribacter*, *Paraprevotella*, and *Akkermansia*, while a decrease in *Blautia* and *Lactobacillus* (Figure 4C). As opposed to Switch 4, Switch 8 showed upregulation of *Akkermansia*, *Odoribactor*, and *Helicobacter*, while *Lactobacillus* and *Dubosiella* manifested downregulation (Figure 4B). To further compare the commonalities of biomarkers discovered by the two methods, we drew an upsets plot. Interestingly, we found that there are intersections in the identification of biomarkers based on the combination of LEfSe and DESeq2 analysis methods. In Switch 4, it appeared as *Dubosella* (Figure S5D). In Switch 8, the occurrences included *Odoribacter*, *GCA_ 900066575*, and *Clostridia_ VadinBB60* (Figure S5E).

### 3.5. Changes and Interactions of Key Bacterium in Switch Group

**Figure 5:**
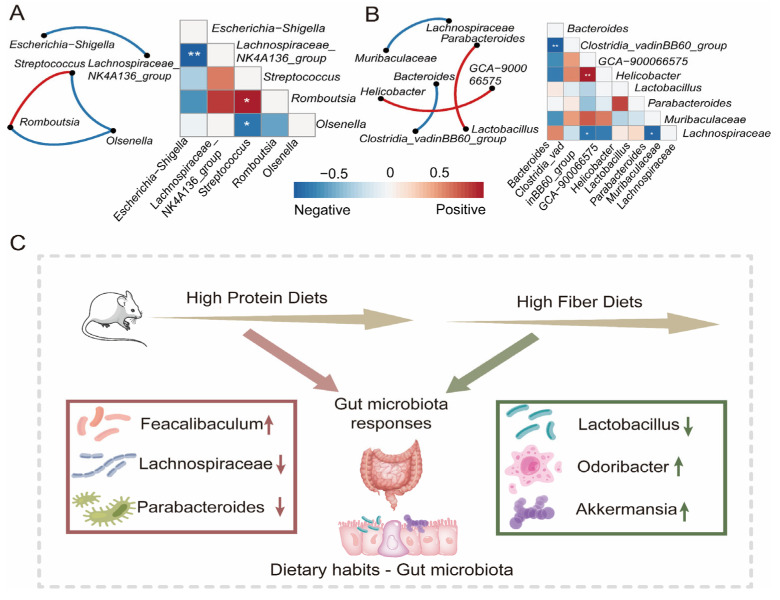
The changes in gut microbiota and the co-occurrence network over time in the Switch group. Correlation and network analysis among the genera of significant difference in the relative abundance were performed in Switch 4 (**A**) and Switch 8 (**B**). The color bar represented the strength of correlation. Edges in different colors between two genera represent the positive or negative correlation. (**C**) A hypothesis model was proposed that HPD affects the composition of gut microbiota, and HFD promotes the recovery of gut microbiota. (* *p* < 0.05, ** *p* < 0.01).

To understand the correlation between gut bacterial composition and the dietary intervention progress in the Switch group, we employed a random forest machine learning algorithm to determine the relative abundance of gut microbiota across the whole research project. We evaluated the importance of genus through cross-validation. In our study, we observed that the cross-validation error curves exhibited relatively low values when employing 17 genera throughout the entire experimental duration (Figure 4D). The abundance of *UCG-010*, *Clostridia_vadinBB60_group*, and *Allobaculum* increased in week 8. *Lactobacillus* significantly decreased after maintaining high abundance for four weeks (Figure 4E). This once again confirms the vital role of key microorganisms in different dietary interventions. To further illustrate the interaction and relationship of genera in terms of changes in relative abundance among groups, we performed Spearman correlation and network analysis. The Spearman correlation calculated the correlation between genera. Subsequently, the genera with significant correlations were used for analyzing interaction networks and to reconstruct the correlation heatmap. The Spearman correlation results indicated similarity in that the complexity of interactions among genera in Switch 4 (Figure 5A) and Switch 8 (Figure 5B). In the Switch 4 and Switch 8 groups, there were fewer significant correlation pairs, and the correlation network was simple. In the Switch 4 group, there was a significant positive correlation between *Streptococcus* and *Romboutsia*. It was worth noting that *Escherichia-Shigella* and *Lachnospiraceae_ NK4A136_ group* had a highly significant negative correlation, which might be closely related to a high protein diet. However, after receiving a high fiber diet, we found a significant positive correlation between *Parabacteroides* and *Lactobacillus* in the Switch 8 group, as well as a significant negative correlation between *Lachnospiraceae* and *Muribaaculaceae*. Consequently, based on the above research, we proposed a hypothesis that a high protein diet would lead to changes in the gut microbial composition and have an impact on gut health, while a high fiber diet can improve the gut microbiota and promote gut health (Figure 5C).

## 4. Discussion

As is widely recognized, the composition of microbiota is related to multiple factors. Dietary habits play a crucial role in altering the microbial composition [28,29]. The correlation between diet and gut microbiota has been demonstrated. The long-term dietary pattern is closely related to the diversity, composition, and related gene content of microbiota [30,31,32,33]. We can better maintain homeostasis and reduce disease susceptibility by introducing dietary habits into the relationship between the host and the microbiota [34]. Poor dietary habits have been proven to be associated with the occurrence and development of non-communicable chronic diseases [35]. The relationship between dietary habits, the plasticity of gut microbiota, and the response of the immune system make microbiota-targeted interventions a focus for disease prevention and treatment [36,37]. Previous studies have indicated a direct relationship between the formation of microbiota and the maternal gut microbiota during the breastfeeding period after newborns are born [38]. However, after weaning, dietary habits become the primary factor influencing the composition of microbiota [39,40]. In this study, we fed early-life mice with a high protein diet and a high fiber diet. The purpose was to explore the dynamic alterations in gut microbiota composition when mice were exposed to these specific dietary patterns for a long-term period.

Our research study had found that consumption of a high fiber diet leads to weight reduction in mice. The significant weight reduction observed in the high fiber group might be a result of lower dietary intake or be related to single nutrient overdose. Research has shown that significant weight loss can be associated with certain conditions, such as inflammatory bowel disease and metabolic disorders [41,42]. Body weight has long been believed to be closely associated with changes in gut microbiota [43]. Nevertheless, we observed that changes in gut microbiota occurred before any noticeable decrease in body weight after consuming a high fiber diet, suggesting that the changes in gut microbiota are caused by the dietary fiber intake. Studies have reported that higher dietary fiber intake is linked with reduced body weight, lower blood cholesterol, and lower systolic blood pressure [44]. Increased intake of dietary fiber is typically associated with an increase in *Prevotella* [45]. Additionally, previous studies showed that when body weight decreases, there is also a decrease in bacteria from the Firmicutes [46]. At the same time, there is an increase in *Akkermansia* and *Faecalibacterium* [47]. These findings align with the results of our study.

Additionally, it was suggested that, compared to the AIN-93G diet, the HFD maintained microbial diversity, but the HPD significantly reduced microbial diversity. Furthermore, we discovered that high fiber diet could restore the diversity of gut microbiota in the Switch group. Previous studies have found a decrease in microbial diversity in patients with inflammatory bowel disease [48,49]. This indicated that a high protein diet poses a threat to intestinal health, and a high fiber diet alleviates this harm. Further research on the Switch group revealed that the acceptance of a high fiber diet resulted in rapid changes in the composition of the gut microbiota. These variations were primarily characterized by a decrease in *Lactobacillus* and an increase in *Akkermansia*. Additionally, the interactions between microorganisms during the period of high protein and high fiber diets were also significantly different. Previous studies on proteins have pointed out that the source and content of proteins can have different effects on gut microbiota [50]. Research has demonstrated that the consumptions of animal protein can lead to an increase in pathogenic bacteria, thereby increasing the susceptibility to inflammatory bowel diseases [51]. Clinical studies indicated that the intake of casein and soy protein can disrupt the normal gene expression of obese people [52]. Conversely, the consumption of dietary fiber helps to maintain the diversity of gut microbiota and promotes the production of short-chain fatty acids through fermentation, thereby safeguarding intestinal health [53].

In our study, we found that in the microbial interaction network of Switch 4, *Escherichia-Shigella* and *Lachnospiraceae_ NK4A136_ group* showed a significant negative correlation, while *Romboutsia* showed a significant positive correlation with *Streptococcus*. *Escherichia-Shigella* is a typical bacterium known for its ability to invade the intestinal tract. It highly adheres to the intestinal mucosa, suppressing the autophagy of inflammatory cells to escape the host’s immune system and induces inflammation, leading to the formation of granulomas in intestinal diseases [54,55]. Numerous studies have identified the presence of *Escherichia-Shigella* in cases of inflammatory bowel disease [56,57,58]. *Lachnospiraceae* is concentrated near the intestinal mucosa and affects the immune function of the intestine by utilizing dietary components to produce short-chain fatty acids [59,60]. It was also found that *Lachnospiraceae* increased and induced the production of butyrate in the study, employing inulin to intervene in ulcerative colitis [61]. *Streptococcus* plays a significant role in the metabolism of amino acids within the intestine. Studies have proved that *Streptococcus* are urea-decomposing bacteria. Through the production of urease, they can break down nitrogen compounds present in dietary ingredients into amine substances. This process can generate toxicity within the intestinal tract [62,63]. *Romboutsia*, a type of probiotic known for its ability to produce butyrate, has been proven to sharply decrease during intestinal mucosal lesions and is considered a potential microbial indicator for the incidence of intestinal diseases [64,65]. Therefore, our results suggest that after the intervention of a high protein diet, pathogenic bacteria become more active, posing a potential threat to intestinal health. However, probiotics that produce short-chain fatty acids show resistance to this damage.

After receiving high fiber diet intervention, the interaction between microbiota was mainly characterized by a positive correlation between *Lactobacillus* and *Parabacteroides*, as well as a negative correlation between *Lachnospiraceae* and *Muribaculacea*. Differential analysis revealed a significant decrease in *Lactobacillus* in Switch 8. This was consistent with previous studies on the fermentation characteristics of pectin in the intestines of piglets, which found that the dominant transformation of *Lactobacillus* after pectin intervention was into *Prevotella* [66]. This is because, under a high fiber diet, *Lactobacillus* has fewer energy sources, resulting in a decrease in abundance. *Lactobacillus* primarily degrades indigestible carbohydrates in the colon through homogeneous or heterogeneous fermentation as its energy source, leading to the production of lactic acid [67]. Research has found that after receiving high fiber diet, the production of secreted IgA is promoted, which can promote the abundance of *Lactobacilli* [68,69]. However, *Lactobacillus* has always been a controversial topic. Most studies have proposed that *Lactobacillus* exist in the intestine as probiotics, but many studies had pointed out that the high abundance of *Lactobacillus* was related to cancer, obesity, and type 2 diabetes [70,71,72,73]. Meanwhile, studies on the gut microbiota of elderly people have revealed a positive correlation between the abundance of *Lactobacillus*, the quantity of white blood cells, and inflammatory factors, such as low-density lipoprotein [74]. Research on enteritis and *Parabacteroides* has demonstrated that this bacterium possesses distinct anti-inflammatory properties. These properties may arise from alterations in the production of local cytokines in the intestine, resulting in the formation of a non-specific anti-inflammatory environment. It can also stabilize the composition of gut microbiota and improve the body’s immune capacity [74]. Our study revealed that the abundance of *Lactobacillus* decreased following the consumption of a high protein diet, and this decrease was likely related to *Paraacteroides*. Their interaction resulted in a competitive relationship, leading to a decrease in *Lactobacillus*. However, it is pivotal to note that this competition promotes the role of a high fiber diet in supporting and protecting the intestinal microbiota for optimal intestinal health.

In addition, *Lachnospiraceae* is the main producer of butyrate [60]. Its emergence can be achieved through efficient ABC transporters, which uptake complex extracellular GH substances and monosaccharides. These substances increase their competitive dietary fiber intake and achieve a protective effect on intestinal health [75,76,77]. Interestingly, *Muribaculacea* has always received attention in shaping the gut microbiota. In the study of gut microbiota in sterile mice, it was found that *Muribaculacea* and *Erysipelotrichaceae* are the core of the gut microbiota [78]. Furthermore, genes related to succinic acid, acetate, and propionate fermentation pathways have been found in the *Muribaculacea* genome. It mainly degrades plant polysaccharides and α-Glucose, etc. [79,80]. This further indicates that after intervention with a high fiber diet, the activity of probiotics that produce short-chain fatty acids increases, promoting the positive effect of diet on intestinal health.

However, we observed that different microbiota played distinct roles at various dietary stages. In Switch 4, *Feacalibaculum* and *Dubosella* were upregulated, while *Parabacteroides* were downregulated. In Switch 8, we found upregulation of *Akkermansia* and *Odoribactor*, but downregulation of *Lactobacillus* and *Romboutsia*. *Feacalibaculum* is commonly recognized as a probiotic that helps to reduce inflammation. It is one of the common butyrate-producing bacteria and is generally believed to play an essential role in cell apoptosis, inflammation, and oxidative stress [48,49]. Nevertheless, the decrease in *Feacalibaculum* in Switch 8 caused an increase in *Akkermansia*. Studies have demonstrated that *Akkermansia* promotes the conversion of tryptophan into indole metabolites in the intestinal microenvironment and plays a protective role in intestinal health [81]. *Akkermansia* has been shown to improve metabolic disorders, inflammatory bowel disease, and colorectal cancer [82,83,84]. Our study indicated that the gut microbiota undergoes rapid changes in response to a high protein diet and high fiber diet, highlighting the robust plasticity of the gut microbiota in response to dietary interventions. These findings are consistent with previous research [34,85]. However, our study revealed that the proportion of Firmicutes and Bacteroidetes (F/B) differed under the influence of different diets. A high protein diet increased the ratio of F/B. The F/B ratio is widely recognized as having a significant impact on maintaining normal intestinal homeostasis [86]. An elevated F/B ratio is closely associated with obesity, a significant public health concern. The gut microbiota plays a role in the occurrence of obesity through direct interaction with proximal organs or indirect interaction with distant organs through metabolites [87]. Adjusting the gut microbiota through dietary modifications, including the supplementation of probiotics and dietary fiber, is an essential approach for treating and preventing obesity [88]. Our study emphasized that the effect of the plasticity of diet on gut microbiota is related to gut health. Diet significantly affects the composition of gut microbiota, and alterations in gut microbiota profoundly impact body health.

In conclusion, our research confirms that dietary habits provide evidence for the plasticity of gut microbiota. We elucidated the changes in gut microbiota under the acceptance of HPD and HFD, and further discussed the correlation between these changes and gut health. Our research suggests that a high protein diet may pose a certain threat to intestinal health. However, after intervention with HFD, there is an increase in probiotics that metabolize short-chain fatty acids, further affecting the interaction between microbiota and hosts, which may have a positive impact on intestinal health. Our study once again emphasizes the impact and plasticity of dietary habits on the gut microbial composition, this alteration that may be closely linked to gut health. 

## Figures and Tables

**Table 1:** Composition of experimental diets.

Ingredient (g/kg)	Control	HPD	HFD
Casein	200	593	181.8
L-Cystine	3	3	3
Corn starch	397	67	377.2
Maltodextrin 10	132	69	120
Sucrose	100	100	90.9
Cellulose	50	50	90.9
Soybean oil	70	70	63.6
T-Butylhydroquinone	0.014	0.014	0.014
Minerals	35	35	24.8
Vitamins	10	10	0.2
Pectin	0	0	45.5
Choline bitartrate	2.5	2.5	2.5

Control, AIN-93G diet [14]; HPD, high protein diet; HFD, high fiber diet.

**Table 2:** Percentage of nutrients and energy in different diets.

Nutrients	Control	HPD	HFD
Carbohydrate	63.2%	23.9%	47.1%
Protein	20%	59.3%	18.2%
Fat	7%	7%	6.4%
Fiber	5%	5%	25.6%
Energy	3.9 Kcal/g	4.0 Kcal/g	3.7 Kcal/g

Control, AIN-93G diet; HPD, high protein diet; HFD, high fiber diet.

## Data Availability

The 16S rRNA Sequencing data reported in this paper have been deposited in the Sequence Read Archive (https://www.ncbi.nlm.nih.gov/sra), under BioProject ID PRJNA972421 (http://www.ncbi.nlm.nih.gov/bioproject/972421).

## Supplementary Materials

The following supporting information can be downloaded at: https://www.mdpi.com/article/10.3390/nu15153460/s1, Figure S1: Alpha dilution curve for all samples (*n* = 60); Figure S2: The weight gain of mice at week 8 and microbial diversity change over time; Figure S3: Beta diversity of different groups analyzed by PCoA; Figure S4: Dynamics of bacterial composition changes with experimental time of four groups at the phylum level; Figure S5: Biomarkers at different experimental time in the Switch group.
