# Supplementary material for: Exploring the Plasticity of Diet on Gut Microbiota and Its Correlation with Gut Health
Source: Nutrients. 2023 Aug 4;15(15):3460. doi: 10.3390/nu15153460 (PMC10420685; doi:10.3390/nu15153460)
Supplement: Supplementary file 1 [file nutrients-15-03460-s001.zip › nutrients-2483434-supplementary.pdf]

Supplementary Material

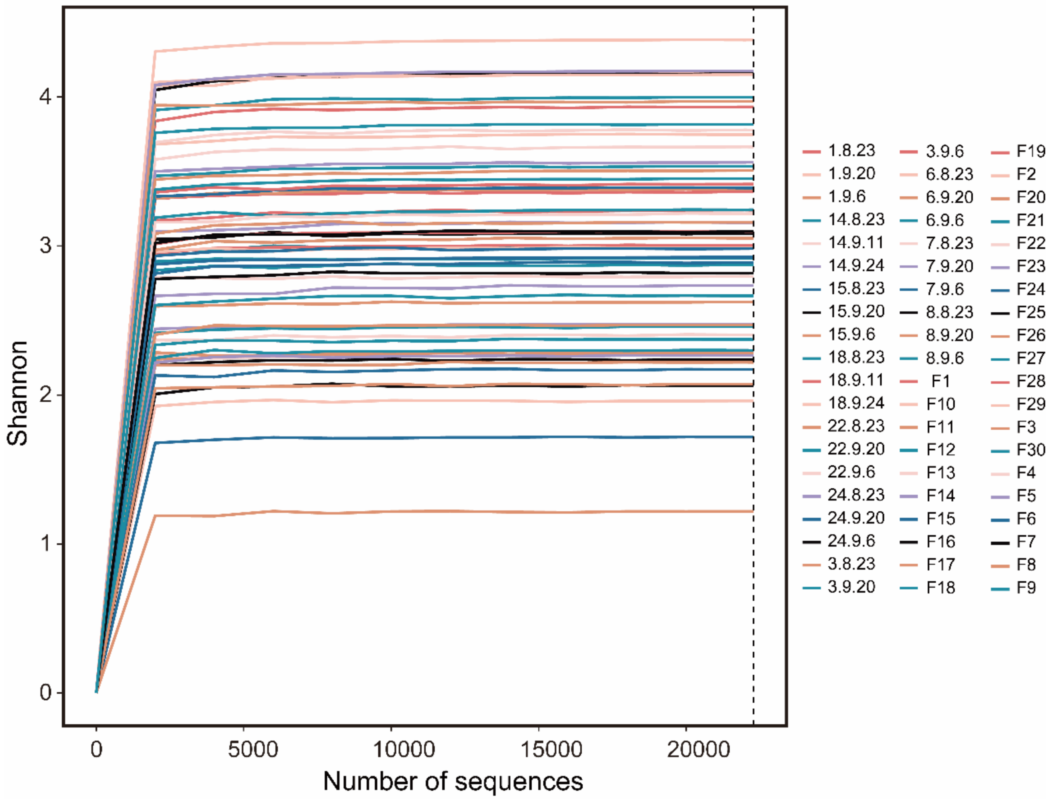

Figure S1. Alpha dilution curve for all samples (n = 60).

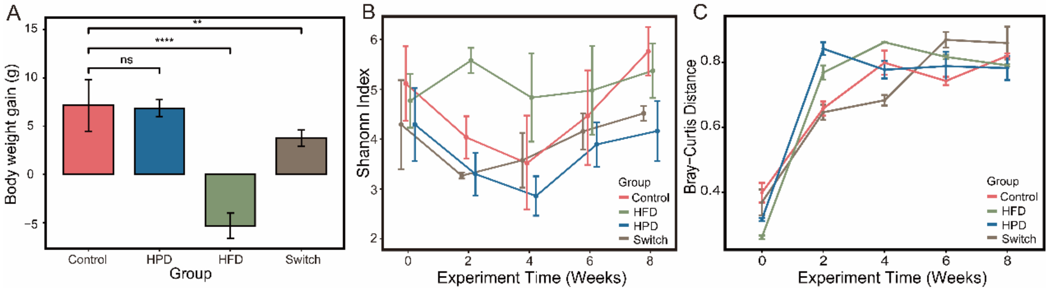

Figure S2. The weight gain of mice at week 8 and microbial diversity change over time. **(A)** The body weight gain of mice at week 8. **(B)** The change of Shannon index over experiment time. **(C)** Bray-Curtis distance displayed change in beta diversity over experimental time. (Wilcox. test, *P < 0.05, **P < 0.01, *** P < 0.001, **** P < 0.0001.)

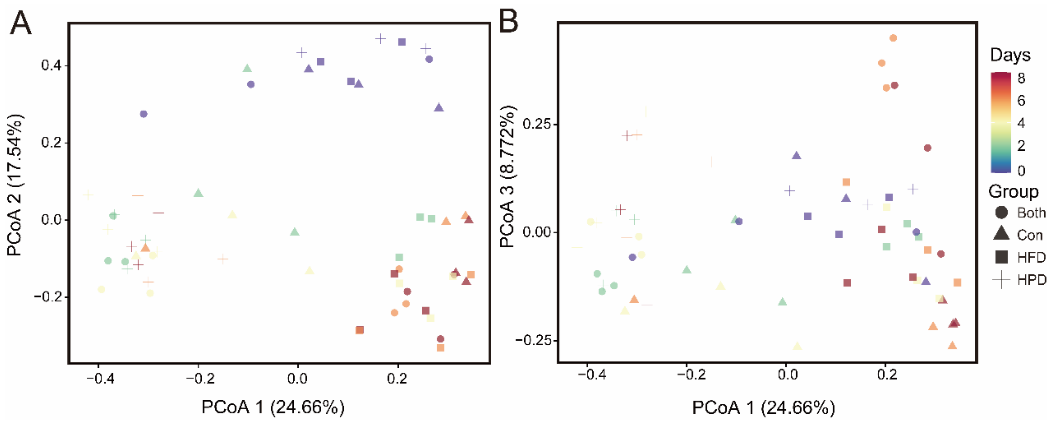

Figure S3. Beta diversity of different groups analyzed by PCoA. (A) Variation of samples on the second coordinate axis. (B) Variation of samples on the third coordinate axis.

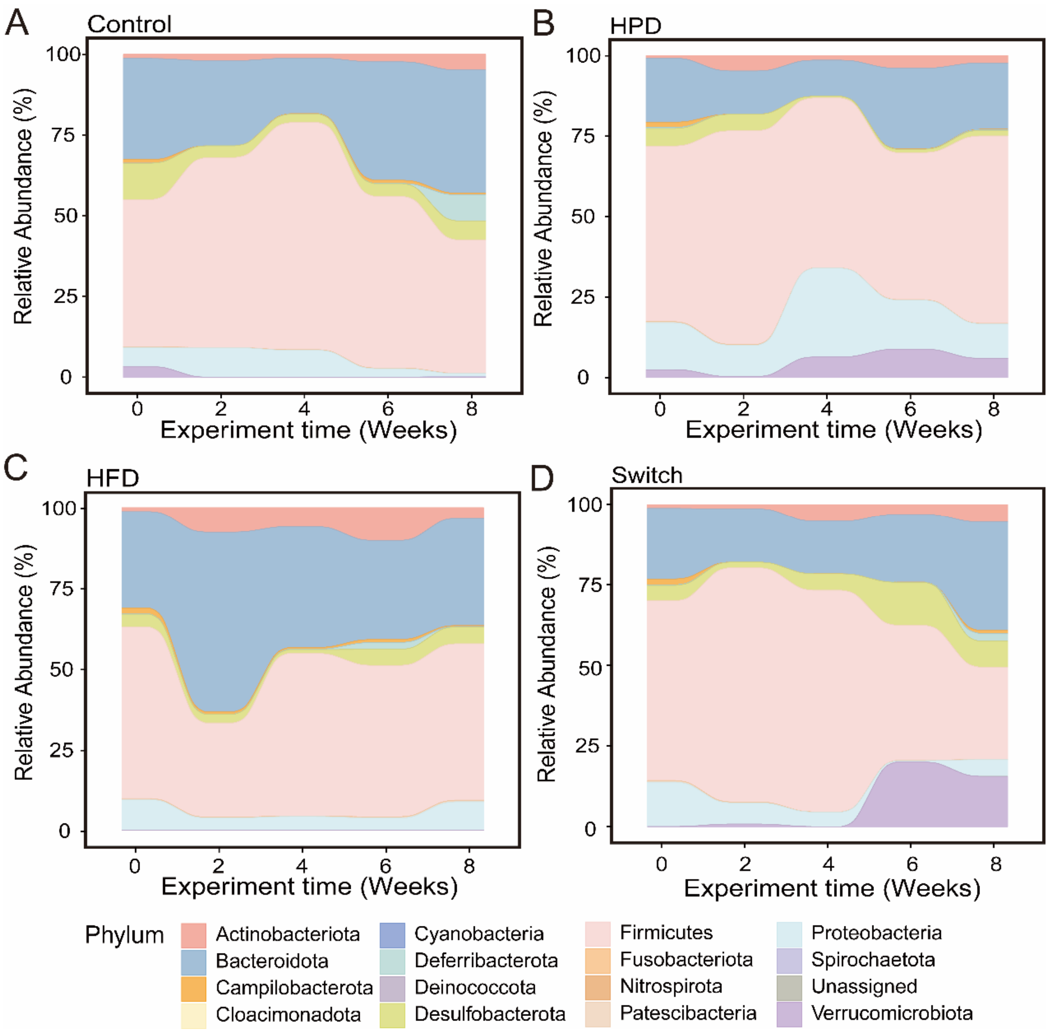

Figure S4. Dynamics of bacterial composition changes with experimental time of four groups at the phylum level. The changes of microbial composition at the phylum level of the Control group **(A)**, HPD group **(B)**, HFD group **(C)**, and Switch group **(D)**.

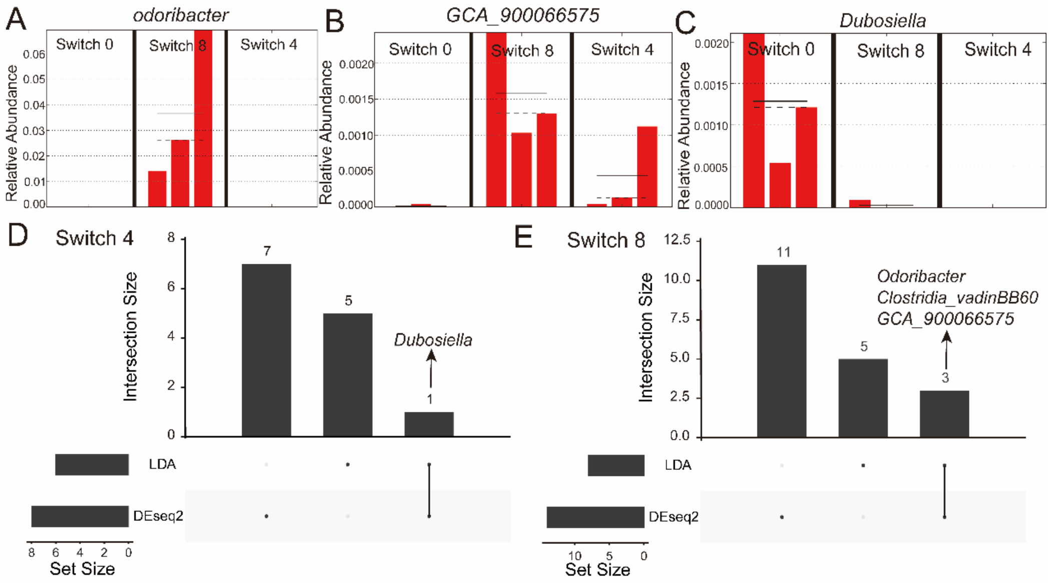

Figure S5. Biomarkers at different experimental time in the Switch group. Comparison of biomarker abundance in each group, *Odoribacter*(**A**), *GCA_900066575*(**B)**, *Dubosiella*(**C)**. This upset plot showed the biomarkers of Switch 4**(D)** and Switch 8**(E)** found based on LDA and DESeq2.
